# Analysis of Mitochondrial DNA Sequences in Childhood Encephalomyopathies Reveals New Disease-Associated Variants

**DOI:** 10.1371/journal.pone.0000942

**Published:** 2007-09-26

**Authors:** Aijaz A. Wani, Sajad H. Ahanger, Sharmila A. Bapat, Ashraf Y. Rangrez, Nitin Hingankar, C. G. Suresh, Shama Barnabas, Milind S. Patole, Yogesh S. Shouche

**Affiliations:** 1 National Centre for Cell Science, Pune, India; 2 Division of Biochemical Sciences, National Chemical Laboratory, Pune, India; North Carolina State University, United States of America

## Abstract

**Background:**

Mitochondrial encephalomyopathies are a heterogeneous group of clinical disorders generally caused due to mutations in either mitochondrial DNA (mtDNA) or nuclear genes encoding oxidative phosphorylation (OXPHOS). We analyzed the mtDNA sequences from a group of 23 pediatric patients with clinical and morphological features of mitochondrial encephalopathies and tried to establish a relationship of identified variants with the disease.

**Methodology/Principle Findings:**

Complete mitochondrial genomes were amplified by PCR and sequenced by automated DNA sequencing. Sequencing data was analyzed by SeqScape software and also confirmed by BLASTn program. Nucleotide sequences were compared with the revised Cambridge reference sequence (CRS) and sequences present in mitochondrial databases. The data obtained shows that a number of known and novel mtDNA variants were associated with the disease. Most of the non-synonymous variants were heteroplasmic (A4136G, A9194G and T11916A) suggesting their possibility of being pathogenic in nature. Some of the missense variants although homoplasmic were showing changes in highly conserved amino acids (T3394C, T3866C, and G9804A) and were previously identified with diseased conditions. Similarly, two other variants found in tRNA genes (G5783A and C8309T) could alter the secondary structure of Cys-tRNA and Lys-tRNA. Most of the variants occurred in single cases; however, a few occurred in more than one case (e.g. G5783A and A10149T).

**Conclusions and Significance:**

The mtDNA variants identified in this study could be the possible cause of mitochondrial encephalomyopathies with childhood onset in the patient group. Our study further strengthens the pathogenic score of known variants previously reported as provisionally pathogenic in mitochondrial diseases. The novel variants found in the present study can be potential candidates for further investigations to establish the relationship between their incidence and role in expressing the disease phenotype. This study will be useful in genetic diagnosis and counseling of mitochondrial diseases in India as well as worldwide.

## Introduction

Mitochondria are keys to many a cellular processes. One of the most important mechanisms is oxidative phosphorylation (OXPHOS) resulting in the production of cellular energy in the form of ATP. The OXPHOS system consists of five multiprotein complexes (I–V) and two mobile electron carriers (coenzyme Q and cytochrome c) embedded in the lipid bilayer of the inner mitochondrial membrane [1], [2]. The mitochondrial genome encodes 13 essential polypeptides of the OXPHOS system and the necessary RNAs machinery (two ribosomal RNAs and 22 transfer RNAs). The remaining structural proteins and those involved in import, assembly and mitochondrial DNA (mtDNA) replication are encoded by the nuclear DNA and are targeted to the mitochondria.

Disorders of mitochondrial origin are a heterogeneous group of diseases commonly manifesting in tissues with high-energy demand, for example, muscle, nerve, and eye. Mutations in respiratory chain protein subunits encoded by either mitochondrial DNA (mtDNA) or nuclear DNA are responsible for such diseases [3]. Mutations in mtDNA are more common than in nuclear DNA because the former mutates 10–17 times faster than later. The reason for high mutation rate is the absence of chromatin and histones. Also the continuous generation of reactive oxygen species (ROS) and the lack of efficient repairing mechanism further increases the mutation rate [4].

Diagnosis of mitochondrial disorders depends on a combination of approaches including clinical analysis, measurement of respiratory chain enzyme activities and morphological analysis. Genetic diagnosis involves the analyses of mutations; mostly those known as primary mutations (disease associated) of mtDNA. Pathogenic mtDNA point mutations are present either in tRNA or in protein coding regions. A number of human genetic diseases of mitochondrial origin have been elucidated [5], [6]. An mtDNA mutation can inherit maternally or arise sporadically. Most of the pathogenic mtDNA mutations are heteroplasmic (a mixture of both mutant and wild type mtDNA in the same cell or tissue) and the disease manifestations or clinical expression depends on the ratio of mutant to wild type mtDNA [7]. However, in case of Leber's hereditary optic neuropathy (LHON) and non-syndromic sensoneural hearing loss, the mutations are almost homoplasmic and only a few cases of heteroplasmy have been observed [8]–[10].

Recent studies have shown that majority of mutations associated with mitochondrial diseases remain unidentified occurring either in mtDNA or nuclear DNA [11]. Mostly the diagnosis is based on primary mutations for diseases like LHON, Leigh's disease, NARP, MELAS etc. However, in majority of the cases, no primary mutations are being detected even if the patient has typical signs of mitochondrial disorder. Recent studies have shown the role of secondary mutations and involvement of other rare or novel mtDNA variants in mitochondrial encephalomyopathies [12]–[17]. The existence of unique and new variants specific to particular populations makes the diagnosis more complicated.

Several researchers are analyzing the complete mtDNA sequences in mitochondrial disorders and have reported a variety of new variants whose mechanism of involvement in the disease is not well established. [13]–[15]. These variants may be found either in protein coding region, in tRNA or very rarely in rRNA genes and are considered to be rare or secondary. A few of these variants have been found in more than one case. Hence, further evidence of these secondary, rare or novel variants is acceptable and it becomes necessary to explore more mtDNA sequences in patients with mitochondrial disorders to establish the relationship of each variant with the disease.

In the present study, we explored the mtDNA sequences of 23 patients (children) with clinical and morphological features of typical mitochondrial dysfunction and efforts were made to establish the relationship of each variant with the disease. Also, this study is aimed to establish a genetic diagnosis mechanism for mitochondriopathies particularly relevant to Indian subcontinent and may also be applicable to other populations.

## Materials and Methods

### Patients and families

A group of 23 pediatric patients clinically diagnosed for mitochondrial disorders were studied. Out of these, 6 were diagnosed with Leigh's syndrome or Leigh's like syndrome, one with MELAS and one with chronic progressive external opthalmoplegia (CPEO). The remaining 15 patients had undefined mitochondrial myopathies or encephalopathies. All the patients had the classical clinical features such as stroke like episodes, neuropathy, seizures, ataxia, optic atrophies, etc. The primary clinical diagnosis was based on high blood and serum lactate levels following the rules of Berner et al. 2002 [18]. No histopathology was done because of not getting the skeletal muscle biopsies due to some ethical issues. The Magnetic Resonance Imaging (MRI) findings were also taken into consideration. Detailed laboratory findings are shown in table 1. Respiratory chain enzyme analysis was done for 14 patients using either primary lymphocytes or established lymphoblast cell lines (Table 2). Majority of the subjects were below the age of 10 years except patient 8 and patient 9 whose ages were 12 and 19 years respectively.

**Table 1:** Laboratory findings of children with mitochondrial disease.

Disorder	Patients	Age (years)	CSF lactate (mg/dl)	Blood Lactate (mg/dl)
*Encephalopathy*	P1	7	28.8	ND
	P3	0.3	22.0	ND
	P4	7	ND	26.2
	P10	4	ND	26.2
	P11	6	14	16.7
	P12	1	47.4	ND
	P15	0.6	High	ND
	P17	9.5	18.7	20.2
	P19	2.5	17.1	29.8
	P20	3	20	ND
	P23	1.8	High	ND
*Leigh's disease*	P2	2.5	46.7	47.5
	P5	0.7	18.25	ND
	P6	4	36.1	ND
	P14	1	Normal	ND
	P18	1	39.4	ND
	P22	7	26.2	ND
*Mitochondrial cytopathy*	P7	1.8	High	ND
	P13	7	26.2	31.0
	P16	2	ND	ND
	P21	7	29	ND
*CPEO*	P8	19	15	ND
*MELAS*	P9	12	26	27

ND = not determined, CSF = cerebrospinal fluid; CPEO = chronic progressive external opthalmoplagia, MELAS = Mitochondrial encephalopathy lactic acidosis and stroke like episodes. Normal range for CSF and Blood lactate is 10.8-18.9 mg/dl.

**Table 2:** Activities of oxidative phosphorylation enzyme complexes in patient and control lymphoblast cell lines.

			Enzyme Complexes			
Patient	I	II	II+III	III	IV	CS
*Patient 1*	9.7*	14	24.33	62.91	333.7	319.43
*Patient 2*	20.28±5.55*	12.78±2.84	22.84±4.08	56.84 ±12.35	227.9±35.48	289.87±8.54
*Patient 3*	28.4	14.1	24.85	59.91	188.7*	293.09
*Patient 4*	24.56±5.74*	12.98±1.36	21.24±2.55	70.57±14.0	372.34±44.88	325.50±12.0
*Patient 5*	12.3*	12.77	23.0	63.10	362.1	328.77
*Patient 6*	13.84±4.36*	12.78±1.64	39.72±6.8	62.78±29.89	299.7± 51**	292.62 ±33.84
*Patient 7*	12.84*	13.0	25.00	35.10*	228.4	322.01
*Patient 9*	8.5*	12.33	23.10	52.33	140.4**	330.41
*Patient 10*	12.84±2.0**	13.36±1.36	24.92±4.08	64.19±12.28	414.00±53.13	317.31±13.0
*Patient 12*	34.0	11.97	23.98	56.10	247.1	299.80
*Patient 13*	10.3*	13.75	24.87	39.13*	235.3	311.23
*Patient 14*	36.0	13.36	25.50	59.10	340.8	301.43
*Patient 19*	8.23*	12.47	26.00	65.10	351.2	318.09
*Controls (n = 3)*	48.40±5.23	12.88±2.42	25.16±4.30	64.40±8.86	379.59±46.81	276.15±15.5

Mitochondrial complex I activity was taken as the one that is rotenone sensitive. Specific enzyme activities shown as nmol/min/mg protein. I; NADH-DB oxidoreductase, II; Succinate-DB oxidoreductase, II+III; Succinate Cytochrome C reductase, IV; Cytochrome C oxidase, CS; Citrate synthase. ** and * indicate *P* values that are stastically significant (p<0. 05 and p<0.005). SD± was calculated for only five cell lines.

The present study was conducted in accordance with the Helsinki Declaration of 1975, as revised in 2000. Blood samples were collected by the expert physicians of three major hospitals in India (namely KEM hospital, Mumbai, Wadia Hospital for children, Mumbai and P.D. Hinduja hospital, Mumbai). A written consent was obtained from parents of all patients included in the study. It is to be noticed that there were no familial relations among the 23 pediatric patients studied.

### Cells

In majority of the patients, primary lymphocytes isolated by Ficoll-Hypaque gradients were used for respiratory chain enzyme (RCE) analysis. However, in 6 patients due to less sample volume available, RCE was performed with lymphoblast cell lines established using Epstein bar virus (EBV) transformation. The cells were maintained in RPMI 1640 medium (Gibco BRL) supplemented with 10% (v/v) heat inactivated fetal bovine serum (Gibco BRL).

### Respiratory Chain enzyme analysis and Rotenone sensitivity

Activities of respiratory chain enzymes viz., NADH dehydrogenase (Complex I), Succinate dehydrogenase (Complex II), Succinate cytochrome C reductase (Complex II+III), and ubiquinol Ferricytochrome C oxidoreductase (Complex III) were determined spectrophotometrically using methods of Trounce et. al [19]. The cytochrome C oxidase (Complex IV) activity was determined by using cytochrome C oxidase kit (Sigma, USA) as per manufacture's instructions. Mitochondrial complex I activity was taken as the rate sensitive to rotenone inhibition (10 µM). Results were expressed as citrate synthase ratios to correct for any differences in sample preparation. For each assay, results are interpreted as the mean of three independent experiments.

### Extraction and PCR amplification of DNA

Total genomic DNA was extracted from the blood using DNeasy tissue kit (Qiagen, Germany). The entire mitochondrial genome was amplified in 1–3 kb overlapping fragments using a subset of 24 primer pairs as described earlier [20]. The PCR reaction mixture consisted of 1× reaction buffer, 2 µM of primers, and 125 µM of dNTPs, 0.1 µg of template DNA and 1 unit of AmpliTaq gold DNA polymerase (Applied Biosystems, USA). The standardized PCR conditions were: initial denaturation at 95°C for 2 minutes, 35 cycles of denaturation at 94°C for 45 seconds, annealing at 60°C for 45 seconds, extension at 72°C for 90 seconds and a final extension at 72°C for 10 min. mtDNA was amplified by 22 overlapping PCR fragments of 800 base pairs to >1 kb. To detect the amplified DNA, 10 µl of PCR product was electrophoresed on a 1% agarose gel and visualized by ethidium bromide staining. The use of large PCR products excluded the possibility that nuclear pseudogenes present will complicate the analysis [21]. The presence of deletions was excluded by long-range PCR (Expand Long PCR, Roche, Germany) in the blood DNA.

### DNA sequencing and Variant identification

Excess primers and dNTPs were removed from the PCR amplified DNA fragments using 0.5 unit of shrimp alkaline phosphatase (Amersham pharamacia, USA) and 2 units of Exonuclease I (Amersham). The fragments were then cycle sequenced using Bigdye terminator cycle sequencing kit with a thermostable thermsequenase II DNA Polymerase. The products were precipitated using salt/ethanol to remove all unincorporated dye labeled terminators, and the pellet was diluted in formamide loading dye and analyzed with an ABI 3730 sequencing instrument (Applied Biosystems, USA). Both forward and reverse primers were used for sequencing.

Sequence data was analyzed for mutations by ABI software (SeqScape version 2.1) and also confirmed by BLASTn program. All the nucleotide sequences were compared with the revised Cambridge reference sequence (CRS) [22] and with those present in two mitochondrial databases; MITOMAP (http://www.mitomap.org) and Human mitochondrial genome database (http://www.genpat.uu.se/mtDB). When the genomic change was located in an encoding region, we used the Mitoanalyzer programme (http://www.cstl.nist) to determine whether the mutation triggered any amino acid change in the polypeptide sequence. All the identified variants were also tested in patient's mothers to find out whether they are maternally inherited or are of de novo origin.

### Analysis of heteroplasmy

**Figure 1:**
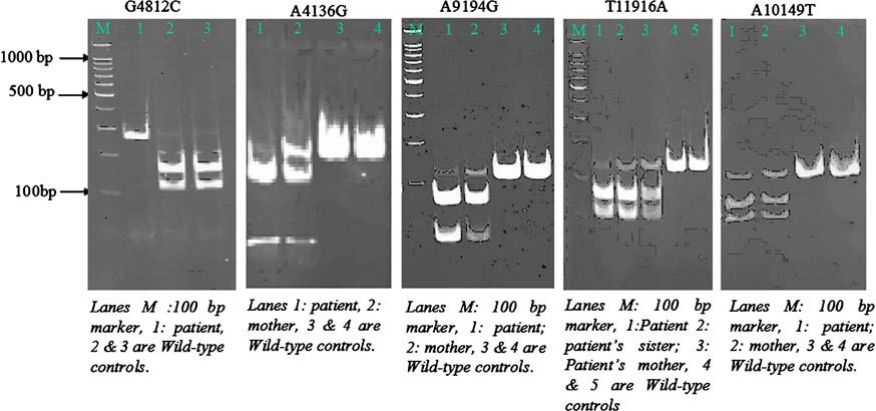
Ethidium Bromide stained polyacrylamide gels of PCR amplified products encompassing heteroplasmic variants G4812C, A4136G, A9194G, A10149T and T11916A analyzed by PCR-RFLP in patients and their respective mothers. Mutation G4812C causes a loss of *Dde I* site and hence shows a single band after digestion whereas wild type fragment shows 2 bands (bp). Variant A4136G creates an *Nla III* site and after digestion results in two bands of 43 bp and 107 bp. The heteroplasmic mutation has three bands (43 bp, 107 bp and 150 bp) as shown. Similarly other variants e.g. A9194G (creates *Hha I* site), A10149T (creates *Dde I* site) and T11916A (creates *Rsa I* site) and all result in 3 bands after digestion and separation on 12% PAGE. The homoplasmic mutations should yield only 2 bands lacking the wildtype band after separation on PAGE.

We considered heteroplasmic variants as those producing double peaks in the electropherograms. All heteroplasmic variants present in protein coding regions, were further confirmed by PCR/RFLP (restriction fragment length polymorphism) analysis. RFLP was performed for only those variants that do create a restriction site. However, some variants did not create any restriction site; hence heteroplasmy for such variants was considered on the basis of double peaks in the sequence electropherograms. For RFLP, amplified DNA was digested in a 50-µl-reaction volume with 5U of restriction enzyme. The mixture was incubated at 37°C for 3 hr, electrophoresed on a 12% Polyacrylamide gel (PAGE) in 2× Tris acetate buffer (TAE) buffer and visualized with ethidium bromide staining (Fig. 1). Enzyme digestion analysis was applied to patients and their respective maternal relatives. Densitometry was used to calculate the amount of wild type and mutated DNA.

### Bioinformatics analysis

To understand the consequences of these mutations upon the structure and interaction of protein subunits, we plotted the hydropathy indices for a moving 9-residue window for both the wild type and mutant polypeptides using Kyte-Doolittle hydropathy plot analysis (http://www.gcat.davidson.edu/rankarin/kytedoolittle.htm) [23]. Kyte-Doolittle plot predicts the possible structure of a protein. Further, the membrane-spanning regions of these proteins were predicted from the SPLIT 4.0 server (http://split.pmfst.hr/split/4/). The purpose of this server is to predict the trasmembrane (TM) secondary structures of membrane proteins using the method of preference functions.

Secondary structures of tRNAs were accessed by using the RNA2 programme at the following website: http://www.genebee.msu.su/services/rna2_reduced.html.

## Results

**Figure 2:**
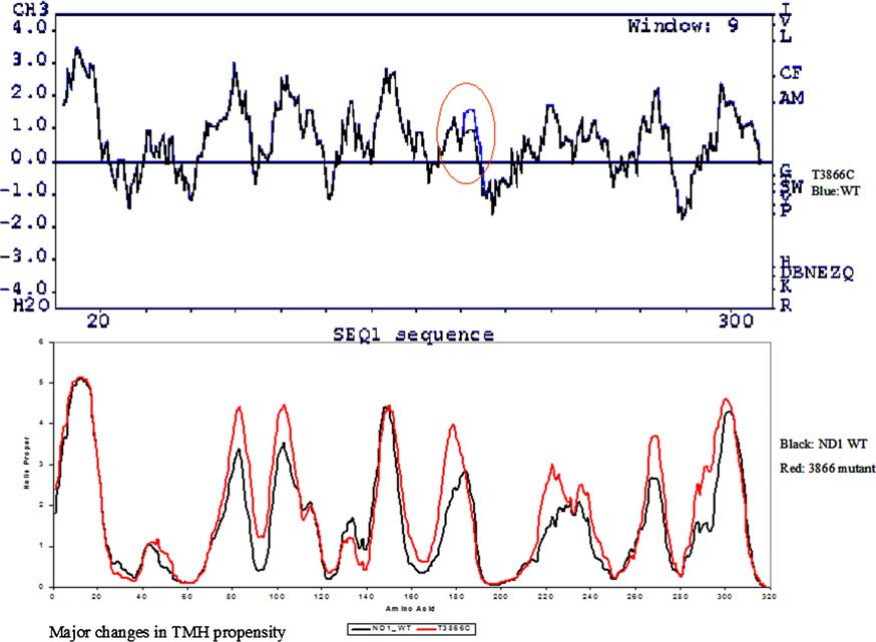
Kyte-Doolittle Hydropathy Plot of wild type and mutated NADH Dehydrogenase Subunits (ND1). Mutation T3866C (Isoleucine-Valine) showing a significant decrease in hydrophobicity at amino acid position 187 in the ND1 protein. SPLIT 4.0 plots predicting Tran membrane regions and alterations in them due to mutation T3866C. Black line is indicative of wild type whereas red line indicates the mutated polypeptide.

We sequenced the mitochondrial genomes of 23 patients with probable encephalopathy and identified a total of 27 significant variants or mutations. Out of these, 12 are novel mutations and remaining 15 are already known mutations associated with mitochondrial diseases (Table 3). Those variants, which do not cause any amino acid change and already reported as polymorphisms in databases, were not considered to be significant and were excluded from further analysis (data not shown). The site of these mutations are as follows: 16 in the protein coding regions (out of which 12 are present in subunits of mitochondrial complex-I genes), 3 in ribosomal RNA, 3 in tRNA coding genes, 2 in complex IV, 1 in cytochrome b and 2 in complex-V genes (ATP synthase). Only one primary disease associated tRNA mutation A3243G was observed in a MELAS patient. Most of the mutations were specifically present in only one case, except G5783A, T4216C and A10149T. Variant G5783A was found in three patients (P7, P13, and P22), whereas variant T4216C (P1 and P3) and variant T10149A (P5 and P8) were found in two cases each. None of the novel variants were found in any of the 105 control mtDNA sequences from Indian population (http://www.mitomap.org and www.genpat.use/mtDB). A few of the known variants viz., T3394C, T3866C, A4136G, T4216C, C4640A, G5460A, G5783A and G9804A (Table 3) were found to be associated with the disease earlier (MITOMAP). Amongst these, variants T3394C, T3866C, A4136G, T4216C and G9804A [in COX III subunit] are considered to be secondary in Leber's hereditary optic neuropathy (LHON) [12], [24]–[26]. Patients harboring variants A4136G, C4640A, and G9804A did not have LHON but other clinical symptoms of mitochondrial disease were evident. The visual acuity of these patients was not affected. Variant T3866C caused extended imbalance in hydrophobicity of mutant ND1 peptide generated with the Kyte-Doolittle algorithm (as evident from its hydropathy plot). This substitution not only substantially reduced the hydrophobicity of extramatrix coil but also influenced the contiguous intra membrane helical domain (Fig. 2). The hydropathy plot was not significantly altered by other variants (data not shown).

**Table 3:** Novel and known mtDNA substitutions in patients with mitochondrial encephalomyopathies.

Patient	Mutation	Gene affected	Amino acid change	Disease association	MITOMAP/mtDB Status
P1	T4216C	ND1	Tyrosine-Histidine	LHON	P. M
	G4812C	ND2	Valine-Leucine	Novel	Not known
P2	T11916A *	ND4	Phenylalanine-Tyrosine	Novel	Not known
P3	T4216C	ND1	Tyrosine-Histidine	Known	P. M
P4	T3866C	ND1	Isoleucine-Threonine	LHON	P. M
P5	2074 del-A	16SrRNA	Not applicable	Novel	Not known
	A10149T	ND3	Methionine-Valine	Novel	Not known
P6	C4640A	ND2	Isoleucine-Methionine	LHON	Provisional
	G15812A	Cytochrome b	Valine-methionine	LHON	Secondary
	15943 del –T	tRNA threonine	Not applicable	Novel	Not known
P7	G5783A *	tRNA cysteine	Not applicable	Myopathy; Deafness	Provisional
P8	A10149T *	ND3	Methionine-leucine	Novel	Not known
P9	A3243G *	tRNA leucine	Not applicable	MELAS	Confirmed pathogenic
P10	T3394C	ND1	Tyrosine-Histidine	LHON/NIDDM	Unclear
P12	G3736A	ND1	Valine-Isoleucine	Novel	Not known
	C8309T*	tRNA lysine	Not applicable	Novel	Not Known
P13	G5783A *	tRNA Cysteine	Not applicable	Myopathy; Deafness	Provisional
P14	A9194G*	ATPase 6	histidine-Aspargine	Novel	Not known
P16	T654G	12S rRNA	Not applicable	Novel	Not known
	T2248C	16S rRNA	Not applicable	Novel	Not known
	A15924G	tRNA threonine	Not applicable	LIMM	P. M
P17	G9804A	COX III	Alanine-threonine	LHON	Provisional
P18	A4704T	ND2	Methionine-Leucine	Novel	Not known
P19	A4136G*	ND1	Tyrosine-Cysteine	LHON	Provisional
P22	G5783A*	tRNA cysteine	Not applicable	Myopathy; Deafness	Provisional
	G7269A	COX I	Valine-methionine	Novel	Not known
P23	T8424C	ATPase 8	leucine-proline	Novel	Not known

Asterisks (*) indicate the variants that are heteroplasmic. ND1, ND2, ND3, ND4 and ND5 are subunits of NADH dehydrogenase (Complex-I). COX-I and COX-III are the subunits of Cytochrome C oxidase (Complex-IV), AD/PD is Alzeimer's Disease and Parkinsons's Disease. Provisional status indicates that only one group has reported the mutation as pathologic. P.M. (point mutation / polymorphism) status indicates that some published reports have determined the mutation to be a non-pathogenic polymorphism. mtDB is Human Mitochondrial genome database (http://www.genpat.uu.se/mtDB).

Variant T4216C occurred to a considerable extent in controls and there are evidences of association of this with LHON (https://www.mitomap.org), however its pathogenicity is not established and there are many controversies. Other homoplasmic variants (e.g. G3736A, A4704T, and G7269A) even though novel did not fulfill all the pathogenicity criteria (not present in MITOMAP and Human mitochondrial genome databases (www.genpat.uu.se/mtDB)). Hence, these might not be significant in terms of pathogenicity and were classified as polymorphisms.

In the present study, some of the variants occurred either singly or in association with other known or novel variants in protein coding or tRNA genes (Table 3). In certain cases, more than one secondary pathogenic variant was found in a single patient (For example, P6 harboring 3 provisionally pathogenic variants: C4640A, G15812A and novel 15943del-T). This typical combination of mtDNA variants (two of them associated with mitochondrial disorders earlier) found in P6 was not found in any of the control mtDNA sequences. This explains that mitochondrial disease can occur due to more than one variant in the mitochondrial genome and may produce a typical clinical phenotype that complicates the diagnosis.

We also observed six heteroplasmic variants viz., G5783A in three cases (P7, P13 and P22), C8309T in one case (P12), G4136A in one case (P19) and T10149A in two cases (P5 and P8). The novel variants T11916A (P2) and A9194G (P14) were found singly. We performed PCR-RFLP analysis for most of the heteroplasmic nucleotide changes (Fig. 1). Unfortunately, due to very less amount of DNA available, we could not estimate the accurate percentage of heteroplasmy by densitometry to repeat the digestion of DNA. Out of these heteroplasmic variants, mutation G5783A in Cys-tRNA and A4136G (Tyr→Cys) are already listed in the MITOMAP. The former being reported from patients with encephalomyopathy and cardiomyopathy [15] and the latter from LHON cases [27]. However, in our study, the patient (P19) harboring A4136G variant did not have LHON but had typical symptoms of mitochondrial disease with brain involvement. Another heteroplasmic variant T10149A was found associated with two cases (Table 3, Fig. 1).

**Figure 3:**
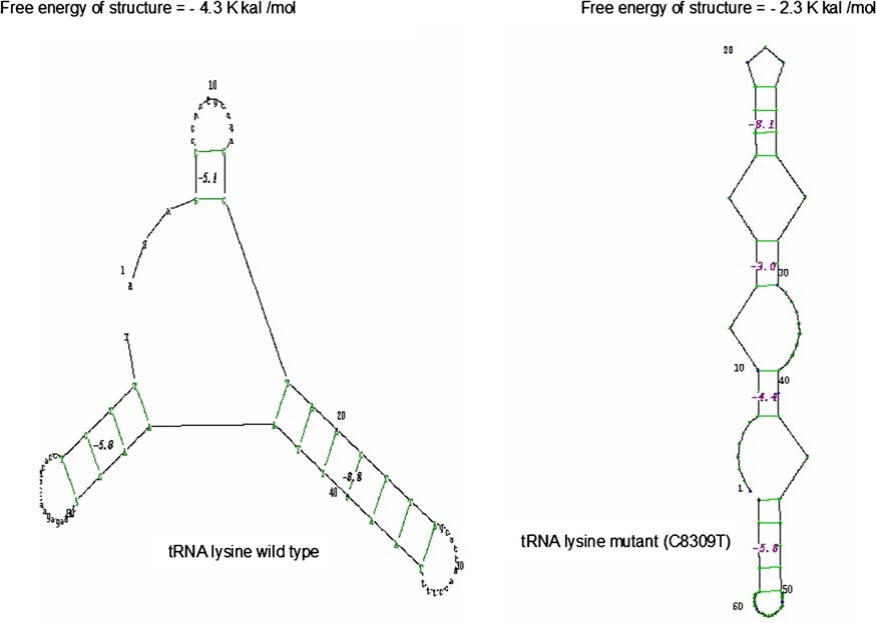
Changes in the secondary structure of tRNA lysine caused by variant C8309T identified in Patient-13. The free energy of the cloverleaf structure is significantly increased in the mutated tRNA lysine.

**Figure 4:**
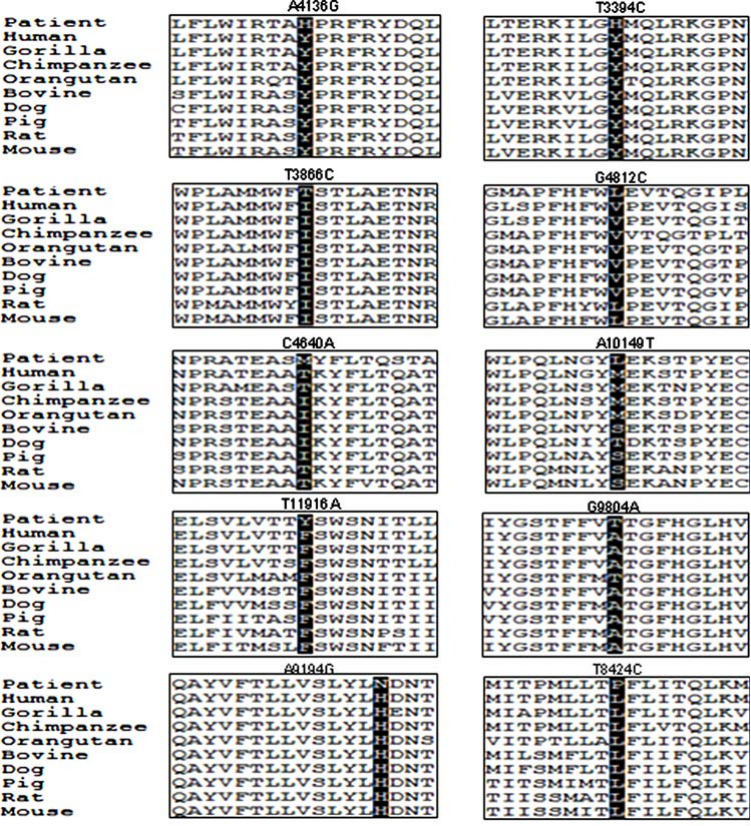
Evolutionary conservation of novel and known amino acid changes in the studied patients. The mtDNA sequences for ND1, ND2, ND3, ND4, COX 3, ATPase 6 and ATPase 8 protein subunits of different mammalian species were converted to protein sequence by Gene Runner software. The amino acid sequences were aligned by Clustal×programme. Accession numbers for the mtDNA sequences used for alignment are: Human (NC_001807), Gorilla (NC_001645), Chimpanzee (NC_001643), Orangutan (NC_001646), Bovine (NC_006853), Dog (NC_002008), Pig (NC_000845), Rat (NC_001665), and Mouse (NC_005089).

Two novel variants were observed in ATPase subunits, one heteroplasmic A9194G in ATP6 (P14) and other homoplasmic T8424C in ATP 8 subunit (P23). The novel tRNA mutation C8309T found in one case (P12) alters the structure of tRNA lysine to a higher extent (Fig. 3). The mutation A9194G changes highly conserved histidine residue to arginine and mutation T8424C changes a conserved leucine to proline (Fig. 4).

All the variants reported in present study were maternally inherited because they were also detected in the mtDNA of their respective mothers. The mothers were asymptomatic and do not express the disease phenotype. However, in some cases (n = 6), the other family members or siblings were also showing the disease symptoms. Moreover, the variants were not located in the pseudogenes of the nuclear genome because the primers used for PCR reaction did not amplify the nuclear product when the DNA from rho zero cells (kindly provided by Dr. Taylor, New Castle, UK) were used.

## Discussion

Complete mitochondrial genome sequencing of patients with childhood encephalopathies allowed us to detect mtDNA variants that can be associated with the disease. We located heteroplasmic mutations in the tRNA genes of five children, one of which is novel C8309T in Lys-tRNA and the other in Cys-tRNA (G5783A) in three patients. One patient had the common MELAS mutation (A3243G). In addition, we found several mutations (A4136G, C4640A, T3394C, G9804A, A9194G) in protein coding region that have been previously reported from LHON and related disorders.

We believe that the mutations A4136G, A9194G and T11916A are the cause of encephalopathy in the patients P18, P2 and P14 respectively. A complex-I deficiency was evident in patients harboring A4136G and T11916A variants (Table 2). These three mutations were absent in 105 healthy control sequences. Alignment of ND1, ND3 and ATPase 6 sequences from various species revealed highly conserved nature of tyrosine; phenylalanine and histidine at these positions in all mammals and other eukaryotes (Fig. 4). Furthermore, these mutations were heteroplasmic in blood which is typical characteristic of pathogenic mutations in mtDNA. The heteroplasmy levels were clear from the RFLP patterns (Fig. 1). The higher percentage of mutant DNA in the proband might explain the severity of clinical phenotypes. However, the pathogenic relevance of variant A10149T is difficult to establish because it is satisfying only a few criteria of pathogenicity such as heteroplasmic nature in blood, a change in moderately-conserved methionine to leucine in ND4 subunit of complex-I (Fig. 4), and is absent in controls. On the other hand, it did not segregate with the clinical phenotype because it occurred in two patients with different clinical findings and its association with rRNA deletion (in P3) or tRNA variant (in P9) might be modulating the phenotype of these two patients. Therefore, A10149T can be classified as a variation of unknown pathogenic relevance. However, our present analysis showed that it does not belong to the common polymorphisms associated with Indian populations [28], [29].

The pathogenic relevance of some homoplasmic variants (T3394C, T3866C, C4640A and G9804A) is also unclear. The patients harboring these mutations did not have LHON but had symptoms of typical childhood encephalopathies. The significance of homoplasmic variations in mtDNA remains uncertain, though several homoplasmic mutations have been implied in disease processes. The high degree of polymorphic variability is a major disadvantage in assessing the pathogenicity of a new base change. In addition, polymorphisms might be relatively rare and might co-segregate with disease, confounding identification of the pathogenic mutation. There are exceptions to the rules of classical pathogenic mtDNA mutations, which are defined as a category of human, maternally inherited disorders characterized by a homoplasmic mtDNA pathogenic mutation with variable penetration and stereotypic clinical expression. These disorders include LHON, mitochondrial non-syndromic sensorineural hearing loss, and a form of mitochondrial hypertrophic cardiomyopathy [8]. Recently, a non-synonymous homoplasmic mitochondrial DNA mutation was reported to be associated with severe COX deficiency, multiple neonatal deaths and Leigh's syndrome [30]. Homoplasmic mitochondrial tRNA variants are considerably under-estimated as a cause of mitochondrial disorders, and they may indeed play a greater role in the development of cardiomyopathy than previously reported [31]. Further analysis of more patients will be helpful to prove the association of T3394C, T3866C, C4640A and G9804A mutations/polymorphisms with mitochondrial diseases. However, the complex I defect in such patients can be possibly because of some unknown mutations in nuclear genes encoding OXPHOS proteins and assembly factors.

Interestingly, we found three tRNA mutations (A3243G, G5783A and C8309T) in 5 out of 23 patients i.e. 21.7% of studied patient group out of which G5783A was present in 3 patients (13.07% of patient group). Thus, it can be confirmed that this mutation is a potential pathogenic mutation. Our results are in agreement with earlier studies which showed mitochondrial tRNA mutations in 18% of encephalomyopathic cases [32].

Given the clinical features and the absence of mtDNA deletions in the above studied patients, the novel as well as known disease associated mtDNA variants which we have identified may well play a direct or indirect role in causing the disease. Two or more variants in the same patient might also have a role in explaining the diversity of clinical phenotypes. In addition, we found many other known polymorphisms which were non-significant and non-pathogenic so were excluded from our analysis. Some of these variants define Indian population specific haplogroup (e.g. A10398G, C10400T) found in 80% of the controls and other mutations (A8860G, A8701G, A4769G, A1438G, A15326G, C7028T, C12705T) were found in nearly 50% of the control mtDNA sequences. However, a different genetic mitochondrial background can further determine the phenotype or as previously known that environmental factors could interact with genetic factors (mitochondrial or nuclear or both) amplifying their effect [33], [34].

In conclusion, complete mitochondrial genome sequencing allowed us to detect both novel and already known variants in children who presented with unexplained encephalopathy and combination of neuromuscular/non-neuromuscular defects with OXPHOS defects. The known mutations identified in this study were already described in association with diseases in one or more cases. Hence, our study further strengthens their involvement in disease and potential pathogenicity which could be correlated in future with diagnosis of the mitochondrial diseases. The novel variants found in present study can be potential candidates for further studies to establish the relationship between their incidence and their role in determining disease. However, we were left with 7 children who did not have any pathogenic (confirmed, provisional or disease-associated polymorphisms) mutations in mtDNA, suggesting that the likelihood of nuclear gene mutations in children with a mitochondrial encephalopathy may be even higher than that of mtDNA mutations. Hence, the effect of nuclear genetic factors or nuclear genes encoding mitochondrial proteins cannot be ignored.

## References

[1] Janssen RJ, van den Heuvel LP, Smeitink JA (2004). Genetic defects in the oxidative phosphorylation (OXPHOS) system.. Expert Rev Mol Diagn.

[2] Smeitink J, van den Heuvel L, DiMauro S (2001). The genetics and pathology of oxidative phosphorylation.. Nat Rev Genet.

[3] Shoffner JM, Bialer MG, Pavlakis SG, Lott M, Kaufman A (1995). Mitochondrial encephalomyopathy associated with a single nucleotide pair deletion in the mitochondrial tRNALeu (UUR) gene.. Neurology.

[4] Martorell L, Segues T, Folch G, Valero J, Joven J (2006). New variants in the mitochondrial genomes of schizophrenic patients.. Eur J Hum Genet.

[5] Simon DK, Johns DR (1999). Mitochondrial disorders: clinical and genetic features.. Annu Rev Med.

[6] DiMauro S, Andreu AL (2000). Mutations in mtDNA: are we scraping the bottom of the barrel?. Brain Pathol.

[7] DiMauro S, Moraes CT (1993). Mitochondrial encephalomyopathies.. Arch Neurol.

[8] Carelli V, Giordano C, d'Amati G (2003). Pathogenic expression of homoplasmic mtDNA mutations needs a complex nuclear-mitochondrial interaction.. Trends Genet.

[9] Hutchin TP, Cortopassi GA (2000). Mitochondrial defects and hearing loss.. Cell Mol Life Sci.

[10] Zhadanov SI, Atamanov VV, Zhadanov NI, Oleinikov OV, Osipova LP (2005). A novel mtDNA ND6 gene mutation associated with LHON in a Caucasian family.. Biochem Biophys Res Commun.

[11] Liang MH, Wong LJ (1998). Yield of mtDNA mutation analysis in 2,000 patients.. Am J Med Genet.

[12] Abu-Amero KK, Bosley TM (2006). Mitochondrial abnormalities in patients with LHON-like optic neuropathies.. Invest Ophthalmol Vis Sci.

[13] Sarzi E, Brown MD, Lebon S, Chretien D, Munnich A (2007). A novel recurrent mitochondrial DNA mutation in ND3 gene is associated with isolated complex I deficiency causing Leigh syndrome and dystonia.. Am J Med Genet A.

[14] Da Pozzo P, Cardaioli E, Radi E, Federico A (2004). Sequence analysis of the complete mitochondrial genome in patients with mitochondrial encephaloneuromyopathies lacking the common pathogenic DNA mutations.. Biochem Biophys Res Commun.

[15] Feigenbaum A, Bai RK, Doherty ES, Kwon H, Tan D (2006). Novel mitochondrial DNA mutations associated with myopathy, cardiomyopathy, renal failure, and deafness.. Am J Med Genet A.

[16] Pereira C, Nogueira C, Barbot C, Tessa A, Soares C (2007). Identification of a new mtDNA mutation (14724G>A) associated with mitochondrial leukoencephalopathy.. Biochem Biophys Res Commun.

[17] Fauser S, Luberichs J, Besch D, Leo-Kottler B (2002). Sequence analysis of the complete mitochondrial genome in patients with Leber's hereditary optic neuropathy lacking the three most common pathogenic DNA mutations.. Biochem Biophys Res Commun.

[18] Bernier FP, Boneh A, Dennett X, Chow CW, Cleary MA (2002). Diagnostic criteria for respiratory chain disorders in adults and children.. Neurology.

[19] Trounce IA, Kim YL, Jun AS, Wallace DC (1996). Assesssment of mitochondrial oxidative phosphorylation in patient muscle biopsies, lymphoblasts and transmitochondrial cell lines,. Methods Enzymol..

[20] Rieder MJ, Taylor SL, Tobe VO, Nickerson DA (1998). Automating the identification of DNA variations using quality-based fluorescence re-sequencing: analysis of the human mitochondrial genome.. Nucleic Acids Res.

[21] Parfait B, Rustin P, Munnich A, Rotig A (1998). Co-amplification of nuclear pseudogenes and assessment of heteroplasmy of mitochondrial DNA mutations.. Biochem Biophys Res Commun.

[22] Andrews RM, Kubacka I, Chinnery PF, Lightowlers RN, Turnbull DM (1999). Reanalysis and revision of the Cambridge reference sequence for human mitochondrial DNA.. Nat Genet.

[23] Kyte, Jack, Doolittle, Russell F (1982). A Simple Method for Displaying the Hydropathic Character of a Protein.. J Mol Biol.

[24] Brown MD, Torroni A, Reckord CL, Wallace DC (1995). Phylogenetic analysis of Leber's hereditary optic neuropathy mitochondrial DNA's indicates multiple independent occurrences of the common mutations.. Hum Mutat.

[25] Brown MD, Voljavec AS, Lott MT, Torroni A, Yang CC (1992). Mitochondrial DNA complex I and III mutations associated with Leber's hereditary optic neuropathy.. Genetics.

[26] Matsumoto M, Hayasaka S, Kadoi C, Hotta Y, Fujiki K (1999). Secondary mutations of mitochondrial DNA in Japanese patients with Leber's hereditary optic neuropathy.. Ophthalmic Genet.

[27] Howell N, Kubacka I, Xu M, McCullough DA (1991). Leber hereditary optic neuropathy: involvement of the mitochondrial ND1 gene and evidence for an intragenic suppressor mutation.. Am J Hum Genet.

[28] Palanichamy MG, Sun C, Agrawal S, Bandelt HJ, Kong QP (2004). Phylogeny of mitochondrial DNA macrohaplogroup N in India based on complete sequencing: implications for the peopling of South Asia.. Am J Hum Genet.

[29] Rajkumar R, Banerjee J, Gunturi HB, Trivedi R, Kashyap VK (2005). Phylogeny and antiquity of M macrohaplogroup inferred from complete mt DNA sequence of Indian specific lineages.. BMC Evol Biol.

[30] McFarland R, Clark KM, Morris AA, Taylor RW, Macphail S (2002). Multiple neonatal deaths due to a homoplasmic mitochondrial DNA mutation.. Nat Genet.

[31] Taylor RW, Giordano C, Davidson MM, d'Amati G, Bain H (2003). A homoplasmic mitochondrial transfer ribonucleic acid mutation as a cause of maternally inherited hypertrophic cardiomyopathy.. J Am Coll Cardiol.

[32] Uusimaa J, Finnila S, Remes AM, Rantala H, Vainionpaa L (2004). Molecular epidemiology of childhood mitochondrial encephalomyopathies in a Finnish population: sequence analysis of entire mtDNA of 17 children reveals heteroplasmic mutations in tRNAArg, tRNAGlu, and tRNALeu(UUR) genes.. Pediatrics.

[33] Marchington DR, Poulton J, Sellar A, Holt IJ (1996). Do sequence variants in the major non-coding region of the mitochondrial genome influence mitochondrial mutations associated with disease?. Hum Mol Genet.

[34] Kovalenko SA, Tanaka M, Yoneda M, Iakovlev AF, Ozawa T (1996). Accumulation of somatic nucleotide substitutions in mitochondrial DNA associated with the 3243 A to G tRNA(leu)(UUR) mutation in encephalomyopathy and cardiomyopathy.. Biochem Biophys Res Commun.

